# A machine-learning approach for predicting the effect of carnitine supplementation on body weight in patients with polycystic ovary syndrome

**DOI:** 10.3389/fnut.2022.851275

**Published:** 2022-08-10

**Authors:** Dong-Dong Wang, Ya-Feng Li, Yi-Zhen Mao, Su-Mei He, Ping Zhu, Qun-Li Wei

**Affiliations:** ^1^Jiangsu Key Laboratory of New Drug Research and Clinical Pharmacy, School of Pharmacy, Xuzhou Medical University, Xuzhou, China; ^2^Department of Pharmacy, Feng Xian People’s Hospital, Xuzhou, China; ^3^School Infirmary, Jiangsu Normal University, Xuzhou, China; ^4^Department of Pharmacy, Suzhou Science & Technology Town Hospital, Gusu School, Nanjing Medical University, Suzhou, China; ^5^Department of Endocrinology, Huaian Hospital of Huaian City, Huaian, China

**Keywords:** machine learning, predicting, carnitine supplementation, body weight, polycystic ovary syndrome

## Abstract

The present study aimed to explore the effect of carnitine supplementation on body weight in patients with polycystic ovary syndrome (PCOS) and predict an appropriate dosage schedule using a machine-learning approach. Data were obtained from literature mining and the rates of body weight change from the initial values were selected as the therapeutic index. The maximal effect (E_max_) model was built up as the machine-learning model. A total of 242 patients with PCOS were included for analysis. In the machine-learning model, the E_max_ of carnitine supplementation on body weight was −3.92%, the ET_50_ was 3.6 weeks, and the treatment times to realize 25%, 50%, 75%, and 80% (plateau) E_max_ of carnitine supplementation on body weight were 1.2, 3.6, 10.8, and 14.4 weeks, respectively. In addition, no significant relationship of dose-response was found in the dosage range of carnitine supplementation used in the present study, indicating the lower limit of carnitine supplementation dosage, 250 mg/day, could be used as a suitable dosage. The present study first explored the effect of carnitine supplementation on body weight in patients with PCOS, and in order to realize the optimal therapeutic effect, carnitine supplementation needs 250 mg/day for at least 14.4 weeks.

## Introduction

Polycystic ovary syndrome (PCOS) is a common endocrine disease, influencing approximately 5–15% of reproductive-age women (1). The clinical characteristics of PCOS include polycystic ovarian morphology, ovulatory dysfunction, and hyperandrogenism (2, 3). The exact mechanism of PCOS has not been elucidated; however, it is clear that the syndrome is associated with metabolic syndrome, obesity, or other comorbidities (4, 5). In recent years, the incidence of PCOS was increasing (6–8), which could cause ratio imbalance of estrogen, insulin resistance, hyperinsulinemia, hyperandrogenemia, endometrial dysfunction, infertility, cardiovascular disease, and obesity (9). In addition, the hormone secretion imbalance of patients with PCOS was usually along with many clinical symptoms, such as menstrual cycle disorders, hirsutism, and obesity, jeopardizing the daily activities of the patients (10–12). The main worldwide reason for female infertility, PCOS, has attracted more and more attention for its features, instead of simply ovarian disease (9). Therefore, the treatment of PCOS has been placed in an increasingly important position.

Carnitine, an essential nutrient in b-oxidation of fatty acids and found mostly in animal foods, such as meat, fish, milk, and dairy products, is the carrier of fatty acids across the inner mitochondrial membrane (13). Many studies had verified that there were potential roles of carnitine in patients with PCOS, such as losing weight and normalizing metabolic profiles (14). Its function is to accelerate fat burning and decomposition by transporting fat to mitochondria. As is known to everyone that carnitine is not a cure for obesity; however, the regulation of PCOS patients’ body weight from carnitine can promote the progress of PCOS treatment. That was to say, the regulation of the body weight of patients with PCOS by carnitine is beneficial to the treatment of PCOS. However, the quantitative effects of carnitine supplementation on body weight in patients with PCOS and optimal treatment scheme are still unknown. Thus, the present study aimed to explore the effects of carnitine supplementation on body weight in patients with PCOS and predict an appropriate dosage schedule using a machine-learning approach.

## Materials and methods

### Enrolled patients

To study the effect of carnitine supplementation on body weight in patients with PCOS, patients with PCOS treated with carnitine supplementation were enrolled from the literature report. The search details are shown in Supplementary Table 1, meantime the inclusion criteria were as follows: (i) patients with PCOS, (ii) patients with the treatment of carnitine supplementation, (iii) patients with body weight information, (iv) patients with exact doses and durations of carnitine supplementation, and (v) patients with the control group. In addition, source, group, carnitine dosage, duration of treatment, body weight, number of people, and age were included in a dataset.

For eliminating the potential baseline effect of different studies, the rates of body weight change from the initial values were selected as the therapeutic index. The computational formula is shown in Equation (a):


(a) $$E\%=\frac{{\text{E}}_{\text{time}}-{\text{E}}_{\text{base}}}{{\text{E}}_{\text{base}}}\,\text{×}\,100\%$$

where E_time_ is body weight value at a time and E_base_ is body weight value at baseline.

### Model building

The maximal effect (E_max_) model built up *via* non-linear mixed-effect modeling (NONMEM, edition 7, ICON Development Solutions, Ellicott City, MD, United States) was used for analysis. In addition, in order to acquire the actual effect of carnitine supplementation on body weight in patients with PCOS, the control effect was needed to be removed from the sum effect, which is shown in Equations (b) and (c):


(b) E=B,i,jE-A,i,jEC,i,j

(c) E=B,i,jEmax,i,j×TimeET50,i,j+Time+εi,jNi,j100

where E_*A, i, j*_ represents the sum effect of carnitine supplementation on body weight in patients with PCOS, such as actual effect and control effect. E_*B, i, j*_ represents the actual effect of carnitine supplementation on body weight in patients with PCOS. E_*C, i, j*_ represents the control effect of carnitine supplementation on body weight in patients with PCOS. i represents different studies and j represents every study’s time point. E_max_ is the carnitine supplementation’s maximal effect on body weight in patients with PCOS. ET_50_ represents the treatment time to achieve half of E_max_ from carnitine supplementation on body weight in patients with PCOS. ε_*i, j*_ represents the study i with j time’s residual error. N_*i, j*_ represents the study i with time point j’s sample size. ε_*i, j*_ is weighted by sample size, which is assumed to be distributed normally, and the distribution characteristic is a mean of 0 and variance of σ^2^/(N_*i, j*_/100).

The exponential error model or additive error model was used for assessing the inter-study variability, which is shown in formulas (d–g):


(d) E=max,i,jE×maxexp(η)1,i

(e) E=max,i,jE+maxη1,i

(f) ET=50,i,jET×50exp(η)2,i

(g) ET=50,i,jET+50η2,i

where η_1, i_ and η_2, i_ represent the variabilities of inter-study, which are added into E_max_ or ET_50_ when available and they are assumed to be distributed normally and the distribution characteristics are a mean of 0 and variance of ω_1, i_^2^ and ω_2, i_^2^, respectively.

The categorical covariates and continuous covariates were assessed using Equations (h) to (j):


(h) P=patP+vCOVar×θcor

(i) P=patP+v(COVar-COV)med⋅θcor

(j) P=patP×v(COVar/COV)medθ⁢cor

Where P_*pat*_ represents the patient’s parameter who was with a covariate value of COVar. P_*v*_ represents the parameter’s typical value. COVar represents the value of covariate. COV_*med*_ represents the covariate’s median value of the population. θ_*cor*_ represents the covariate’s correction coefficient with the model parameter.

The potential covariates included source, carnitine dosage, initial body weight, and age, when the basic model was built up, potential covariates were considered to add into E_max_ or ET_50_, respectively. If one or more potential covariables were included in E_max_ or ET_50_, they indicated that one or several covariables would affect efficacy through influencing E_max_ or ET_50_. If no potential covariable was included, it meant that none of the current potential covariables would affect efficacy. The changes from the objective function value (OFV) were selected for the criteria of covariate inclusion. If the OFV value’s reduction was more than 3.84 (χ^2^, α = 0.05, degrees of freedom (d.f.) = 1), the corresponding covariable met the inclusion criteria. If the increase of OFV value was more than 6.63 (χ^2^, α = 0.01, d.f. = 1), the corresponding covariable met the criteria, adding to the final model (15).

### Model evaluation

The final model was evaluated using goodness-of-fit plots [individual predictions vs. observations, conditional-weighted residuals (WRES) vs. time] and the distribution of conditional WRES for the model (quantiles of conditional WRES vs. quantiles of normal). The bootstrap’s median values and 2.5–97.5% values were used for comparing to the final model parameter values. In addition, the predictive performance of the final model was assessed by prediction-corrected visual predictive check (VPC) plots.

### Model prediction

The final model’s E_max_ efficacy curve included the time of duration required to achieve 25%, 50%, 75%, and 80% (plateau) E_max_ of carnitine supplementation on body weight in patients with PCOS were simulated using the Monte Carlo method.

## Results

### Included patients

A total of 242 patients with PCOS were included for modeling (16–19). The carnitine dosage was from 250 to 1,000 mg/day, and the duration of treatment was 12 weeks. The mean age range of patients with PCOS was from 23.6 to 30.8 years. The change rates of body weight from baseline in the included studies were from −1.97 to −3.88%. Supplementary Figure 1 is a strategy for literature search and Supplementary Table 2 shows the detailed patients included in the analysis. The body weight change of the studied subjects post-carnitine administration is shown in Supplementary Figure 2.

### Model building

In the final model of machine learning, the E_max_ of carnitine supplementation on body weight was −3.92%, and the ET_50_ was 3.6 weeks, in addition, no covariate was included in the final model.

The final model is shown in formula (k):


(k) $$E=\frac{-3.92\%\times \text{Time}}{3.6+\mathrm{Time}}$$

Where E represents the effect of carnitine supplementation on body weight in patients with PCOS. The time represents carnitine supplementation treatment duration in patients with PCOS.

### Model evaluation

**FIGURE 1:**
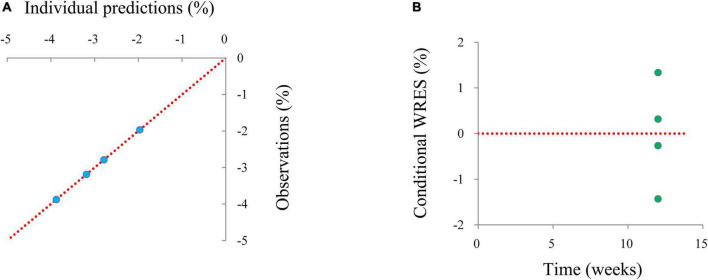
The goodness-of-fit plots of a model. **(A)** Individual predictions vs. observations, **(B)** conditional-weighted residuals (WRES) vs. time.

**FIGURE 2:**
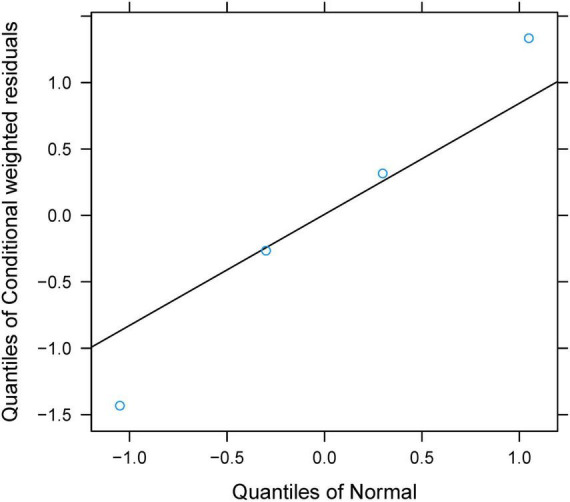
The distribution of conditional-weighted residuals (WRES) for a model.

**FIGURE 3:**
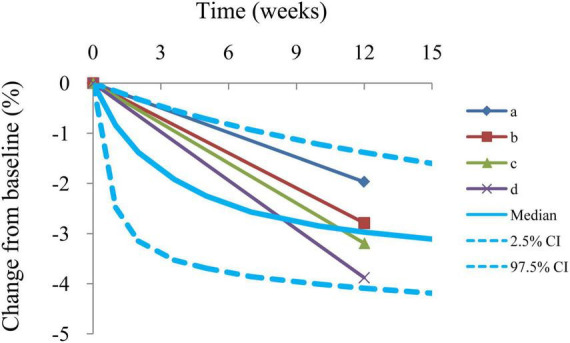
Prediction-corrected visual predictive check plots. Median, 2.5% CI, and 97.5% CI were simulated by Monte Carlo (*n* = 1,000); CI, confidence interval. a–d: 4 groups from included studies (16–19).

Individual predictions vs. observations and conditional WRES vs. time are shown in Figure 1, and quantiles of conditional WRES vs. quantiles of normal are shown in Figure 2, indicating better correlativity in individual predictions and observations. Figure 3 shows the prediction-corrected VPC plots, where most observed data fell within the 95% confidence interval (CI) generated by model simulations, showing that the final model had good forecasting ability. Table 1 shows the final model’s parameter estimated values and bootstrap, where the 1,000 bootstrap’s median values are near to the corresponding final model’s parameter values, showing that the final model was accurate and reliable.

**TABLE 1:** Parameter estimates of the final model and 95% confidential interval.

Parameter	Estimate	Simulation (*n* = 1000)		Bias
		
		Median	95% confidence interval	
E_max_, %	−3.92	−3.92	[−3.92, −3.92]	0
ET_50_, weeks	3.6	3.6	[0.297, 9.89]	0
ω_Emax_	0.106	0.098	[0.003, 0.339]	−0.075
ω_ET50_	0.867	0.376	[0.003, 2.117]	−0.566
ε	0.01	0.01	[0.01, 0.01]	0

95% confidential interval is shown with 2.5th, 97.5th percentile; E_max_ is the maximal effect; ET_50_ is the treatment duration to reach half of E_max_; ω_Emax_ is the inter-study variability of E_max_; ω_ET50_ is the inter-study variability of ET_50_; and ε is the residual error; bias = (median-estimate)/estimate.

### Model prediction

**FIGURE 4:**
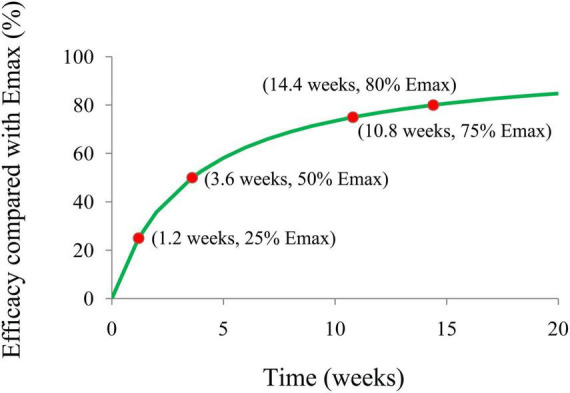
Model prediction.

Figure 4 is the efficacy trend from carnitine supplementation on body weight in patients with PCOS, where the treatment times to achieve 25%, 50%, 75%, and 80% (plateau) E_max_ of carnitine supplementation on body weight are 1.2, 3.6, 10.8, and 14.4 weeks, respectively. In addition, no covariate was included in the model. No significant relationship of dose-response was found in the dosage range of carnitine supplementation used in this study whose carnitine dosage was from 250 to 1,000 mg/day, indicating the lower limit of carnitine supplementation dosage, 250 mg/day, could be used as a suitable dosage. In other words, in order to realize the ideal therapeutic effect, carnitine supplementation needs 250 mg/day for at least 14.4 weeks.

## Discussion

Carnitine, whose structural formula is an essential water-soluble molecule (20), is one of the quaternary ammonium cationic complexes with two stereoisomers (bioactive L-carnitine and abiotic enantiomeric isomer D-carnitine) (21), among which the main carnitine available in the medical and biological domain is L-carnitine, which is also called “carnitine” (21), and whose quality in the human body is approximately 300 mg/kg (21, 22). D-carnitine without bioactivities in people, however, has adverse biological effects *via* suppressing the carnitine acetyltransferase (23), with supplementation of D-carnitine inducing oxidative stress, liver inflammation, and apoptosis in animal studies (24), causing secondary carnitine deficiency, and therefore, almost no human studies of D-carnitine have been conducted (21).

At present, carnitine has many functions, including reducing oxidative stress, increasing expressions of pro-inflammatory cytokines (25–27), improving mitochondrial dysfunction (28) and insulin resistance (29, 30). Therefore, based on these effects, carnitine has an increasingly key role in treating some illnesses. For example, it was found that carnitine as a supplement may be used to treat diabetes mellitus (29, 30), liver cirrhosis (31), dyslipidemia (32), hypertension (33), heart failure (34), coronary artery disease (35), non-alcoholic fatty liver disease (36), Alzheimer’s disease (37), migraine (38), and muscle injury (39). In addition, carnitine treatment of PCOS had been reported in many pieces of literature, for example, according to the current evidence, Maleki et al. found that carnitine might improve weight loss, glycemic status, and oxidative stress (14). Salehpour et al. found that treatment with carnitine in patients with PCOS might decrease the risk of cardiovascular events by normalizing metabolic profiles and body mass index (BMI) using self-controlled study before and after treatment (40). Liao et al. found that carnitine supplementation in women with PCOS had beneficial effects on weight loss and lipid profiles (41). However, the quantitative effects of carnitine supplementation on body weight in patients with PCOS and optimal treatment scheme are still unknown. Therefore, the present study aimed to explore the effect of carnitine supplementation on body weight in patients with PCOS and predict an appropriate dosage schedule using a machine-learning approach.

Machine learning was one of the artificial intelligence fields, which could be used for solving medical problems *via* building up computational modeling based on data. Compared with traditional statistical analysis, machine learning had better behavior (42, 43). Nowadays, machine learning is widely used in the medical field, for example, Tsang et al. reported a pilot study of machine-learning-based automated planning for primary brain tumors (44). Wang et al. reported predicting the targets of interferon regulatory factor-8 (IRF8) and nuclear factor-activated T cells c1 (NFATc1) during osteoclast differentiation using the machine-learning method framework cohort-based TF target prediction system (cTAP) (45). Ohanyan et al. reported that machine-learning approaches to characterize the obesogenic urban exposome (46). Ku et al. reported machine-learning models for predicting the occurrence of respiratory diseases using climatic and air-pollution factors (47). Zeng et al. reported developing a machine-learning model to predict severe chronic obstructive pulmonary disease exacerbations: a retrospective cohort study (48). Ebrahimi et al. reported predictive determinants of overall survival among re-infected patients with COVID-19 using the elastic-net regularized Cox proportional hazards model: a machine-learning algorithm (49). Together, these studies further supported the feasibility of machine-learning approaches to problem solving.

In the present study, data were obtained from literature mining, and the rates of body weight change from the initial values were selected as the therapeutic index. The E_max_ model built up *via* NONMEM was used as the machine-learning model. Weight reduction in kilograms was not taken into account, because the initial body weight of patients with PCOS varied in several of the studies included. Therefore, to eliminate the potential baseline effect of different studies, the rates of body weight change from the initial values were selected as the therapeutic index, which was a better indicator used to assess changes in body weight. In addition, as was known to everyone, the calorie deficit diet, Mediterranean diet, or vegetarian diet may affect weight change. Thus, the carnitine group included in this study had its corresponding control group, so the non-carnitine effect of the control group was deducted from the carnitine treatment group mainly included the effect from the diet, etc. In other words, in order to acquire the actual effect of carnitine supplementation on body weight in patients with PCOS, the control effect needs to be removed from the sum effect. Certainly, this method had been applied in many previous studies (50, 51). Through this scheme, the influence of different diets in different studies could be eliminated. More importantly, the actual effect of carnitine on the body weight of patients with PCOS could be obtained.

In addition, in the four studies included in the analysis, the diagnosis of PCOS was made according to the Rotterdam criteria. A total of 242 patients with PCOS were included for modeling (16–19). In the machine-learning model, the E_max_ of carnitine supplementation on body weight was − 3.92%, the ET_50_ was 3.6 weeks, and the treatment times to achieve 25%, 50%, 75%, and 80% (plateau) E_max_ of carnitine supplementation on body weight were 1.2, 3.6, 10.8, and 14.4 weeks, respectively. The potential covariates included source, carnitine dosage, initial body weight, and age. If one or more potential covariables could be included in E_max_ or ET_50_, indicating that one or several covariables would affect efficacy through influencing E_max_ or ET_50_. If no potential covariable was included, it meant that none of the current potential covariables would affect efficacy. The final model found that no covariate was included in the final model. No significant relationship of dose-response was found in the dosage range of carnitine supplementation used in this study whose carnitine dosage was from 250 to 1,000 mg/day, indicating the lower limit of carnitine supplementation dosage, 250 mg/day, could be used as a suitable dosage. In other words, in order to realize the ideal therapeutic effect, carnitine supplementation needs 250 mg/day for at least 14.4 weeks.

However, this study also has some limitations. First of all, the study population was mainly from Iran, and the number of participants was small, so it needs to be further verified by increasing the sample size in future studies.

## Conclusion

The present study explored the effect of carnitine supplementation on body weight in patients with PCOS using machine learning for the first time, and in order to realize the optimal therapeutic effect, carnitine supplementation needs 250 mg/day for at least 14.4 weeks.

## Data availability statement

The original contributions presented in this study are included in the article/Supplementary material, further inquiries can be directed to the corresponding authors.

## Author contributions

D-DW, S-MH, PZ, and Q-LW conceived and designed the study. D-DW, Y-FL, Y-ZM, S-MH, PZ, and Q-LW collected the data. D-DW built the model, evaluated the data, and wrote the manuscript. All authors read and approved the manuscript.
