# Supplementary material for: A machine-learning approach for predicting the effect of carnitine supplementation on body weight in patients with polycystic ovary syndrome
Source: Front Nutr. 2022 Aug 10;9:851275. doi: 10.3389/fnut.2022.851275 (PMC9399747; doi:10.3389/fnut.2022.851275)
Supplement: Supplementary file 1 [file Data_Sheet_1.docx]

**Table 1S. Search details**

| No. | Query | Results |
| --- | --- | --- |
| #1 | Carnitine[Title]) | 7,685 |
| #2 | L-carnitine[Title] | 2,882 |
| #3 | Levo-carnitine[Title] | 12 |
| #4 | Acetyl carnitine[Title] | 42 |
| #5 | Acetyl L-carnitine[Title] | 562 |
| #6 | ACAL[Title] | 2 |
| #7 | Propionyl L-carnitine[Title] | 150 |
| #8 | PLC[Title] | 850 |
| #9 | Polycystic ovary syndrome[Title] | 9,842 |
| #10 | PCOS[Title] | 1,877 |
| #11 | Syndrome of polycystic ovary[Title] | 10,142 |
| #12 | Stein Leventhal syndrome[Title] | 500 |
| #13 | Polycystic ovary disease[Title] | 73 |
| #14 | PCOD[Title] | 12 |
| #15 | #1 OR #2 OR #3 OR #4 OR #5 OR #6 OR #7 OR #8 | 8,535 |
| #16 | #9 OR #10 OR #11 OR #12 OR #13 OR #14 | 12,087 |
| #17 | #15 AND #16 | 20 |

**
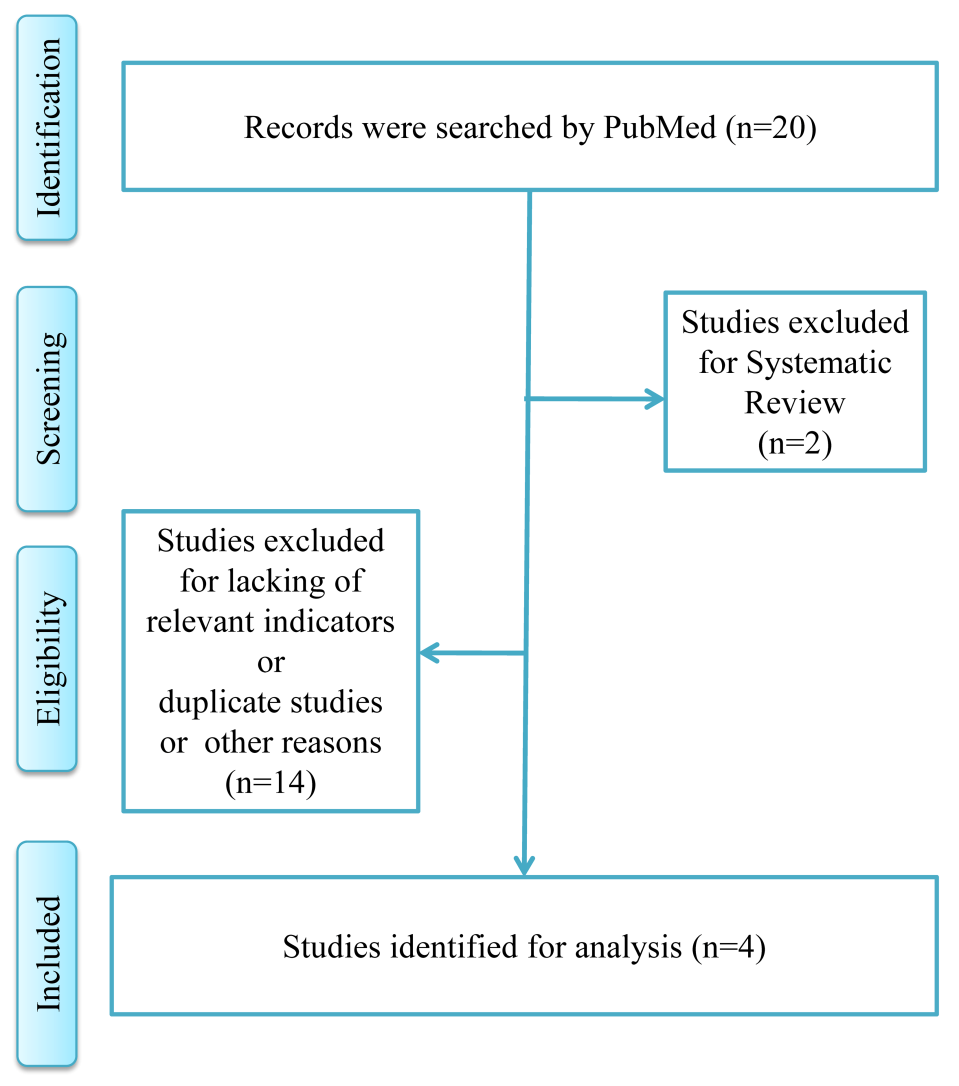
**

**Figure 1S. Strategy for literature search**

**List of final included studies (n=4)**

1: Sangouni AA, Pakravanfar F, Ghadiri-Anari A, Nadjarzadeh A, Fallahzadeh H, Hosseinzadeh M. The effect of L-carnitine supplementation on insulin resistance, sex hormone-binding globulin and lipid profile in overweight/obese women with polycystic ovary syndrome: a randomized clinical trial. Eur J Nutr. 2021 Nov 2. doi: 10.1007/s00394-021-02659-0. Epub ahead of print. PMID: 34727201.

2: Talari HR, Azad ZJ, Hamidian Y, Samimi M, Gilasi HR, Ebrahimi Afshar F, Ostadmohammadi V, Asemi Z. Effects of Carnitine Administration on Carotid Intima-media Thickness and Inflammatory Factors in Patients with Polycystic Ovary Syndrome: A Randomized, Double-blind, Placebo-controlled Trial. Int J Prev Med. 2019 Jun 7;10:89. doi: 10.4103/ijpvm.IJPVM_2_18. PMID: 31360336; PMCID: PMC6592103.

3: Jamilian H, Jamilian M, Samimi M, Afshar Ebrahimi F, Rahimi M, Bahmani F, Aghababayan S, Kouhi M, Shahabbaspour S, Asemi Z. Oral carnitine supplementation influences mental health parameters and biomarkers of oxidative stress in women with polycystic ovary syndrome: a randomized, double-blind, placebo-controlled trial. Gynecol Endocrinol. 2017 Jun;33(6):442-447. doi: 10.1080/09513590.2017.1290071. Epub 2017 Feb 21. PMID: 28277138.

4: Samimi M, Jamilian M, Ebrahimi FA, Rahimi M, Tajbakhsh B, Asemi Z. Oral carnitine supplementation reduces body weight and insulin resistance in women with polycystic ovary syndrome: a randomized, double-blind, placebo-controlled trial. Clin Endocrinol (Oxf). 2016 Jun;84(6):851-7. doi: 10.1111/cen.13003. Epub 2016 Jan 29. PMID: 26666519.

**Table2S. Studies identified for analysis**

| Studies | Sources | Groups | Carnitine  (mg/day) | Duration of treatment (weeks) | Body weight  (kg) | Number of people | Age  (years) |
| --- | --- | --- | --- | --- | --- | --- | --- |
| Sangouni AA (2021) | Iran | Carnitine | 1000 | 12 | 81.4±13.0 | 31 | 30.7±6.7 |
|  |  | Control | - | 12 | 82.2±12.0 | 31 | 30.8±6.6 |
| Talari HR (2019) | Iran | Carnitine | 250 | 12 | 75.1±9.4 | 30 | 23.6±4.6 |
|  |  | Control | - | 12 | 74.3±17.3 | 30 | 25.0±5.4 |
| Jamilian H (2017) | Iran | Carnitine | 250 | 12 | 75.3±8.7 | 30 | 27.1±5.2 |
|  |  | Control | - | 12 | 75.4±13.0 | 30 | 27.2±5.1 |
| Samimi M (2016) | Iran | Carnitine | 250 | 12 | 72.2 ±10.2 | 30 | 24.8±5.5 |
|  |  | Control | - | 12 | 72.9±11.0 | 30 | 25.5±5.7 |


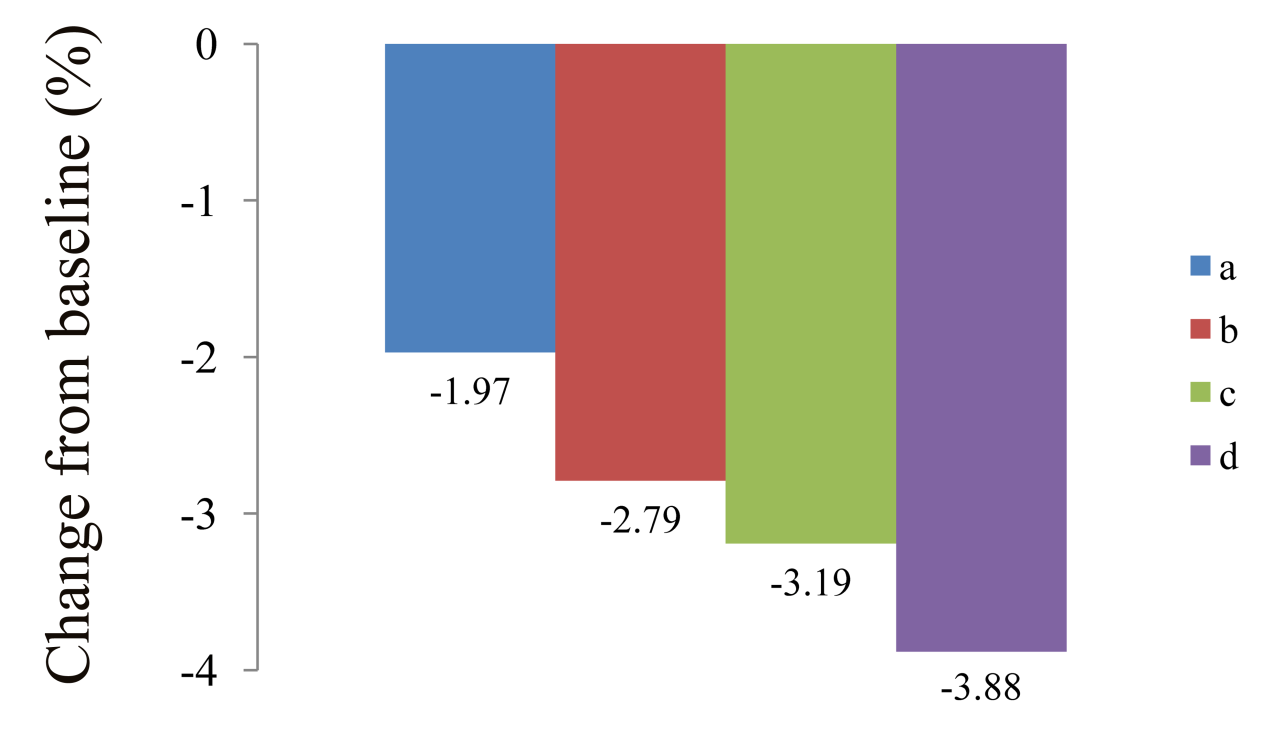


**Figure 2S. Change from baseline in the included studies**

a-d were from included studies([Samimi et al., 2016](#_ENREF_37); [Jamilian et al., 2017](#_ENREF_15); [Talari et al., 2019](#_ENREF_40); [Sangouni et al., 2021](#_ENREF_38)).
